# Development of a Tool for Reporting Key Dietary Indicators from Sales Data in Remote Australian Aboriginal and Torres Strait Islander Community Stores

**DOI:** 10.3390/nu16071058

**Published:** 2024-04-04

**Authors:** Emma McMahon, Megan Ferguson, Thomas Wycherley, Anthony Gunther, Julie Brimblecombe

**Affiliations:** 1 Wellbeing and Preventable Chronic Disease Division, Menzies School of Health Research, Charles Darwin University, Darwin, NT 0810, Australiaanthony.gunther@menzies.edu.au (A.G.);; 2 Department of Nutrition, Dietetics & Food, Monash University, Notting Hill, VIC 3168, Australia; 3 School of Public Health, University of Queensland, Herston, QLD 4006, Australia; 4 Alliance for Research in Exercise, Nutrition and Activity (ARENA), University of South Australia, Adelaide, SA 5001, Australia

**Keywords:** nutrition, dietary indicators, dietary assessment, Aboriginal and Torres Strait Islander, food retail, sales data, information systems

## Abstract

Reporting key dietary indicators from sales data can help us guide store decision makers in developing effective store policy to support healthier customer purchases. We aimed to develop a web-based reporting tool of key dietary indicators from sales data to support health-promoting policy and practice in stores in geographically remote Aboriginal and Torres Strait Islander communities. Tool development included identifying key dietary indicators (informed by sales data from 31 stores), community consultation (19 Aboriginal and Torres Strait Islander store directors and two store managers) and a web-build. Tool evaluation involved feedback interviews with stakeholders (25 store managers and two nutritionists). Key dietary indicators aligned with Australian Dietary Guideline food groupings and recommendations. An online portal for accessing and customising reports was built. Stakeholder feedback indicated that the strengths of the reports were the visuals, ease of interpretation, providing information that was not currently available and potential to increase capacity to support healthy food retailing. Difficulties were defining healthiness classification with alignment to other nutrition guidelines used and ensuring reports reached relevant store decision makers. This tool may be valuable to support store decision makers in identifying and prioritising nutrition issues and optimising the health-enabling attributes of stores.

## 1. Introduction

Improving population diet is a public health priority globally [1]. Retail food stores, as the primary source of food in many settings, can have substantial influence on dietary intake [2,3]. There has been increasing interest from policy makers, public health advocates and retailers in how retail food stores can be optimised to support healthy food choice [2,3,4,5,6,7]. Indicators from sales data can be valuable in identifying the strategies most likely to be effective for improving nutrition outcomes and can also be used to monitor the implementation and evaluate the effectiveness of such strategies [8,9,10,11]. Researchers working with the food retail sector in remote Aboriginal and Torres Strait Islander communities of Australia have demonstrated the power of these data to inform local health-enabling food retail policy [12,13,14].

In Australia, approximately one-fifth of Aboriginal and Torres Strait Islander Peoples live in remote or very remote areas in small towns commonly referred to as communities and/or homelands [15]. Remote community stores are often the primary source of food and other goods in these communities, and they may provide other essential services (e.g., postal, banking, phone access) and/or be a social centre of the community. Remote community stores are often owned by the community and governed by a community-elected group (store directors, store board or store committee) who have not only commercial responsibilities but often also an explicit social responsibility to improve food security, nutrition and health for the communities they serve [16,17,18]. Remote store decision makers, including Aboriginal and Torres Strait Islander store directors and retailers, have demonstrated their ability and willingness to be drivers of change through developing and implementing health-promoting policy and practice [13,14,16,17,18,19].

In 2008, Brimblecombe et al. developed the Remote Indigenous Stores and Takeaways Keeping Track of Healthy Food tool (RIST tool), which aimed to provide timely and relevant information to remote store decision makers on key nutrition indicators from sales data for planning, evaluation and policy decision-making [12]. The RIST tool comprises a set of indicators developed with consideration of the Australian Dietary Guidelines [20], key dietary concerns identified from sales data provided by six remote communities and input through an expert committee [12]. The intent was for the tool to be used by stakeholders such as store boards and retailers as part of a progressive cycle of planning, action and reassessment for improved nutritional quality of foods available through remote community stores. The RIST tool semi-automates the upload and analysis of sales data from food retailers to provide a visual indicator report. Testing of the tool demonstrated its functionality and potential usefulness to monitor and set store performance targets and provide valuable feedback to store boards and other community leaders [12]. Recommendations for future use of this tool were to establish a framework for its implementation, establish performance targets (long-term and intermediary), consider the development of a web-based tool to optimise user experience and minimise technical issues, enhance its accessibility and review indicator relevance abreast with food supply changes and emerging evidence [12].

A modified version of the RIST tool was used with the Good Food Systems Good Food for All project, which used a participatory continuous quality improvement approach to increase the availability, affordability and accessibility of healthy food, developed in 2009–2013 with four remote communities and other stakeholders [13]. In this project, quarterly reports were generated on an abbreviated set of indicators determined as important to monitor by the participating communities. These included absolute and relative store-level month-by-month sales of sugar-sweetened beverages, fruit and vegetables, confectionery and the ten top-selling product lines, as well as proportional dollar spend of different food groups. The multi-sector food interest groups (including store board directors and community leaders) who used these reports emphasised their usefulness in providing feedback on community nutrition and their impact on the performance of some indicators as part of a structured continuous improvement approach [13]. All communities requested the reports to be displayed in their community store and made routinely available [13,19].

We aimed to build from this previous research by developing a web-based reporting tool of key dietary indicators from sales data for use by key stakeholders of stores in remote communities, including Aboriginal and Torres Strait Islander store boards, retailers and store/public health nutritionists, to support health-promoting policy and practice. In developing this tool, we considered the recommendations for improvements to the RIST tool through (i) employment of a web-based platform which may aid accessibility and minimise technical issues while allowing for a broader range of visualisation options than those previously available; (ii) developing key dietary indicators that are relevant to current food supply and purchases (informed by an extended and recent store sales dataset); and (iii) incorporating comparator values alongside key dietary indicators, such as dietary guideline targets and average values across all stores. In this paper we describe the tool development process, providing an overview of the reports developed and their evaluation using feedback from store managers and store nutritionists.

## 2. Materials and Methods

### 2.1. Study Design

Tool development involved an iterative, staged process including identifying key dietary indicators (February to June 2017), community consultation (March to May 2017) and web-build (February 2017 to April 2018). Tool evaluation used qualitative methods involving interviews with stakeholders (April to October 2018) to collect feedback on use of the reports and their value from a user perspective. This study was approved by the Human Research Ethics Committee of the Northern Territory (HREC 2016-2592; Approval Date: 4 July 2016) and Central Australian Human Research Ethics Committee (HREC-16-399; Approval Date: 9 May 16). The authors of this paper are non-Indigenous with extensive experience working with remote stores in Aboriginal and Torres Strait Islander communities.

### 2.2. Setting

There are over 200 stores in remote Aboriginal and Torres Strait Islander communities in Australia, with over half located in the Northern Territory [21]. These stores service communities that range in population size from <250 to >2500 [21]. There is great diversity of languages, cultures, ways of life and kinship structures among these communities. Due to their remoteness, access to services can be limited. Food is freighted by road transport, sea barge, small aircraft or all three.

Approximately half of remote stores across Australia are managed and/or owned by a store management company (store group), with the other half being independently managed (privately or by an Indigenous corporation) [21]. The Arnhem Land Progress Aboriginal Corporation (ALPA) and Outback Stores (OBS) are two large remote retail store groups in Australia. ALPA is an Aboriginal-owned organisation governed by an Aboriginal Board of Directors comprised of representatives from five member communities [22]. ALPA owns/manages 26 community stores in the Northern Territory (NT) and Queensland (QLD) [22]. Outback Stores is a Commonwealth government statutory entity with an independent board that offers a retail management service to 54 community stores across Australia [16]. ALPA and OBS stores are community-owned and have Aboriginal and Torres Islander store boards that meet quarterly. Both these store groups employ nutritionists (store nutritionists). 

### 2.3. Participants

Participants were remote stores in Aboriginal and Torres Strait Islander communities. We aimed to recruit at least 30 stores that represented the different remote store governance models (managed independently; managed by store groups) from a convenience sample of stores with which we had pre-existing relationships. Consent to provide store sales data was provided by store owners (i.e., community store boards when community-owned, or business owners when not community-owned). Participants for community consultation and feedback interviews were drawn from this sample.

### 2.4. Tool Development

#### 2.4.1. Store Sales Data

All participating stores provided weekly product points of sales data showing quantity purchased and dollar value for each product sold in each week from January 2015 to June 2017. Using established methods for translating weekly store sales data into food and nutrient estimates [11,12], store sales data were linked to food and nutrient composition information using the Australian Food Supplement and Nutrient (AUSNUT) database [23], the Australian Dietary Guidelines Database [24] and the Discretionary Food List developed by the Australian Bureau of Statistics [25]. These food and nutrient estimates from store sales data were used to inform the key dietary indicators presented in reports to stakeholders prior to stakeholder feedback.

#### 2.4.2. Key Dietary Indicators

The project team met regularly, drawing from our previous tools [11,12], experience working with remote stores and communities (including reporting nutrition indicators from sales data), values from sales data, food and nutrient databases [23,24,25] and evidence for healthy diets [20,26,27,28,29,30,31] to reach consensus on key dietary indicators, including (i) categories of foods to report on, (ii) disaggregation into subgroups, (iii) categorisation according to healthiness, (iv) numerator (dollars, weight, serves) and denominator and (v) development of reference values. We considered the following principles when identifying and defining key dietary indicators: (i) meaningfulness (as proxy indicators of diet quality), (ii) comparability (over time and across stores), (iii) understandability (stakeholders can make judgments on how the indicators reflect diet quality), (iv) completeness (indicators represent a broad range of products available in store) and (v) relevance (indicators reflect products commonly purchased in communities, using sales data).

#### 2.4.3. Community Consultation

A convenience sample of three communities was selected for community consultation, aiming for a subsample that represented the geographical diversity of the broader sample. In a visit to each of the three communities, we outlined the aims of the project to participants and, using visual aids, sought input/feedback on clarity and cultural appropriateness of the following: (i) visual data options, (ii) descriptors for healthy/unhealthy food group categories, (iii) food categories and iv) colour choices. Responses were scribed during the meeting and summarised descriptively.

#### 2.4.4. Web-Build

An online portal (“FoodFox”) where users can access, customise and download their store reports was built by a commercial webpage developer, and explanatory documents were developed.

### 2.5. Tool Evaluation

#### Feedback Interviews

We aimed to recruit store managers from all participating stores and nutritionists from all participating store groups for feedback interviews. Participating stores were sent paper and electronic copies of the store-specific login credentials, FoodFox reports and explanatory documents (frequently asked questions and serve size guide) in April 2018. All participating stores were contacted via phone from May to July 2018 to seek feedback from store managers on the FoodFox reports. At interview start, store managers were asked if they had had an opportunity to look at the reports and were given the option to reschedule if they had not. The following questions were asked: “Have you had an opportunity to share this information with the store board?”, “Is there any support or training that you think would be helpful for using these reports? (if so, what?)”, “How often do you think it would be useful to receive these reports?”, “How do these reports add value to the information the store board is already receiving?” and “Do you have any other comments or questions?”. The two store nutritionists were asked the same questions and also “What is the best way to disseminate reports to key people within the organisation?”.

Responses to questions were scribed during phone calls, and each participant was emailed their transcript and provided with the opportunity to modify their responses to ensure that the scribed responses were consistent with the intended meaning. Data were managed in NVivo (version 12), coded to the questions and summarised descriptively or quantified where appropriate.

Verbal consent was obtained before proceeding with phone interviews, and participants were asked to return written consent. Twenty-five store managers and both nutritionists returned their written consent forms and were included in the analysis.

## 3. Results

### 3.1. Participants

The 31 participating stores were located in the Northern Territory (25 stores) or Northern Queensland (6 stores) and were mostly managed by ALPA (47%; 15 stores) or Outback Stores (31%; 10 stores), with the remainder managed and/or owned independently or by small store groups (19%; 6 stores). Most stores were governed by an Aboriginal and Torres Strait Islander Store Board (69%; 22 stores).

Community consultation occurred with 21 people (19 store board directors and two store managers) from the communities where three of the participating stores were located.

Evaluation feedback interviews were conducted with store managers from the 31 participating stores and the two nutritionists from store groups (ALPA and Outback Stores).

### 3.2. Tool Development

#### 3.2.1. Key Dietary Indicators

**Figure 1:**
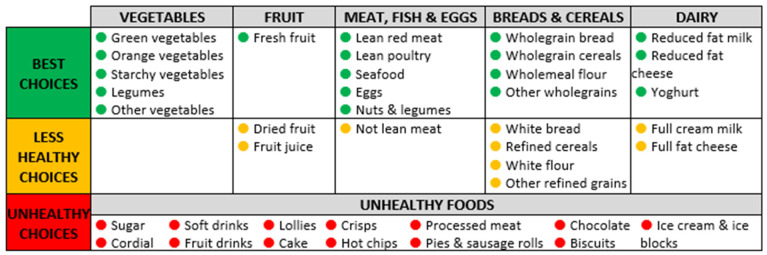
Key dietary indicators: food groups and subgroups.

Figure 1 shows key dietary indicators, including food groups, subgroups and categorisation according to healthiness. Key dietary indicators were those aligned with the Five Food Groups (Fruit; Vegetables; Breads and Cereals; Meat, Fish and Eggs; Dairy); and discretionary foods (Unhealthy Foods) of the Australian Dietary Guidelines (meaningful in terms of diet quality; completeness). These key indicators were aggregated into three subgroups (Best Choices, Less Healthy Choices, Unhealthy Choices) as per Figure 1. The Australian Dietary Guidelines Database [24] was used as a data source when building indicators from the Five Food Groups. Discretionary foods (unhealthy foods) were identified using the Australian Bureau of Statistics Discretionary Food List [25]. The Australian Dietary Guidelines Database gives the option for these to count towards the Five Food Groups (e.g., the potato in deep-fried hot chips can contribute towards vegetables); however, discretionary foods were excluded from contributing to the Five Food Group indicators to avoid confusion (understandability) and to be consistent with the dietary guidelines (meaningfulness as proxy for diet quality). This is consistent with the approach used by the Australian Bureau of Statistics in the Australian Health Survey [32]. The Australian Health Survey Classification System [24,33] was used to categorise discretionary foods into subgroups. For practical purposes, the biggest contributors to discretionary energy were included as subgroups (relevance). Figure 1 shows these 13 subgroups, which combined represented 87.7% of all discretionary energy from all purchased foods and drink products when store sales data from the 31 participating stores were pooled (range for individual stores was from 83.5% to 92.1% of all discretionary energy purchased). Alcoholic beverages were excluded from sales data due to the variation in availability of alcohol from the store and/or other vendors in the community (comparability across stores).

The numerator chosen for reporting was number of serves, as this directly links to Australian Dietary Guidelines targets (meaningfulness as proxy for diet quality), which provide recommendations as serves per person per day. Energy was used as a denominator to allow for comparison to be made across stores and over time (comparability).

As Australian Dietary Guideline recommendations are given as serves/person/day according to population subgroups (e.g., age, gender), we calculated mean reference values based on the proportion of the population in each of these subgroups according to 2011 Census data (Indigenous Australian population living in very remote parts of Australia) [34]. We calculated the average estimated energy requirement to be 8.9 MJ/person/day and used this as a denominator to enable comparison to reference values. Therefore, the reports provide the number of serves of each of the six food groups (and subgroups) purchased per 8.9 MJ of energy purchased (average daily energy requirement), reported as serves/person/day for ease of interpretation. Table 1 shows reference values (targets) calculated using the above method (rounded to the nearest 0.5 serves). The upper limits of discretionary food recommendations were used to derive targets, even though these are only for very tall or active people, because the values from store sales data were two to three times greater than recommendations (relevance).

#### 3.2.2. Community Consultation

**Figure 2:**
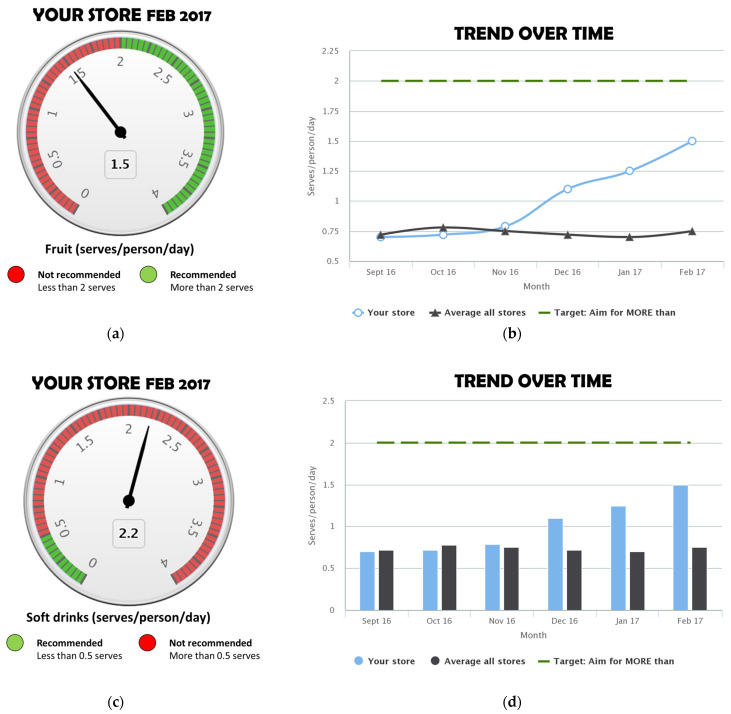
Example visuals: (**a**) gauge chart (healthy food type); (**b**) line graph; (**c**) gauge chart (unhealthy food type); (**d**) bar graph.

Chart type: Some participants preferred the gauge graph (Figure 2a,c) over a bar chart, stating that the similarity to a speedometer made it recognisable and that the use of red and green colours made it easy to see whether the store was meeting the target. Others found the gauge graph confusing at first and said it needed more explanation; specific feedback regarding this was for more detail on what a serve was. To show trend over time, line (Figure 2b) and bar (Figure 2d) charts were both well received; some participants indicated that these were also used in other types of reports they receive. The line chart was preferred by some participants to better show change over time.

Food group descriptors: There were several preferred descriptors for healthiness categories, and those that were consistently preferred and aligned well with each other were “Best Choice”, “Less Healthy Choice” and “Unhealthy Foods”.

Colour: There was consensus that traffic light colours were appropriate for representing the healthiness of foods.

Other food categories: When asked about other foods to include in reports, participants in two communities referred to bush foods (also known as traditional foods).

#### 3.2.3. Web-Build

Preferred visuals (gauge graph for showing current value versus target, line graph for trend over time) and wording for food group descriptors as identified in community consultation were incorporated into reports. Supplement S1 shows an example FoodFox report created using mock data. Supplement S2 shows the explanatory document (frequently asked questions and serve size guide). The online portal included customisation options related to time period (number of months or quarters to display), comparators (average of all stores, dietary guideline targets, user set targets) and key dietary indicators (with the option to select or unselect for reports).

### 3.3. Feedback Interviews

During the initial phone call, most of the store managers (56%; *n* = 14) indicated that they had not looked at the reports. It was common for store managers to state that they were relatively new to the store or had recently returned (e.g., from leave). None of the store managers had shared the information with their store board, with many saying that there had not been a quarterly board meeting since they received the reports or, as above, that they were new to the store or had been away. Some said they would share them at an upcoming meeting, and several mentioned the potential value of sharing the reports with others, e.g., store staff, area managers or the health clinic.

Most store managers (60%; n = 15) reported they and/or their store boards were currently receiving some sort of nutrition-related reports (mostly those owned/serviced by ALPA or Outback Stores), and others indicated they had received such information in the past (but were not currently receiving nutrition-related reports). The types of information described were percentage of dollars spent on product categories such as fruit and vegetables, drinks, fresh meat and/or table sugar, as well as reports on availability of specific products. All store managers that reported receiving some sort of nutrition-related reports indicated that the FoodFox reports added value to the information they were already receiving—for example, by providing more comprehensive information about a larger range of food groups, more detail within food groups and information that is clearly linked to health.

*“they go into more detail (otherwise [we receive information on] just sugar, fruit, veg, soft drinks)”*

*“The reports break down the food groups into more detail (e.g., green vegetables, orange vegetables). They are detailed and good, and also align with activities by the health clinic who do work on healthy eating and bring out people to talk about food and cooking. All of these information sources tie in together well.”*

*“useful to have outside reports to show a different perspective”*

In general, store managers indicated that they liked the visual presentation of information, that it was easy to see at a glance, that they liked the reporting of many types of food groups and types of foods within these groups and that the reports showed healthy versus unhealthy foods, as well as showing comparisons to targets or the average of all stores to benchmark against.

*“The reports help to define what categories aren’t in the target zone (for good health) so can work on getting them into the target zone.”*

*“The good thing is that it shows the best choices and the less healthy choices. Clearly defines there subgroups related to health, e.g., fruit fresh (best choices) versus dried fruit and fruit juice (less healthy choices).”*

*“…useful to see change. For example, if I moved fruit and veg to a more prominent position to try to increase sales—the reports showing before and after the move would be useful to see change.”*

Some store managers saw the reports as one part of taking a holistic approach to enabling healthy food purchasing.

*“The reports do add value, best as part of the whole process where the customers are educated on cooking, budgets, etc. and the reports help to be able to see shifts in consumer purchasing.”*

Timeliness of reports was considered important. Store managers indicated that quarterly or half-yearly reports would be most useful to align with other reporting timelines or with store board meetings.

Store managers reported no support or training was needed for using the reports, describing the reports as being “*straight-forward”* and *“easy to understand”* and stating that the ways the data were displayed were consistent with other types of reports they currently receive or had received previously. Some suggested that more information on the purpose of the reports and how the data were obtained would be useful.

The store group nutritionists indicated that the reports were useful and valuable to their role in supporting stores and that they added value to the current types of data available, including reporting on a wider variety of foods and types of food and reporting of measures directly related to healthy diets (i.e., serves rather than dollar sales).

*“The value add from [FoodFox] reports are by reporting on the amount purchased rather than dollar sales. Prices change so reporting on dollar sales doesn’t provide a full picture, and doesn’t always reflect a change in quantity, e.g., if mangoes prices went up, an increase in dollar sales might not mean we are selling more. With fruit and vegetables, we are trying to report on quantity, but because of the way it is in the system it is difficult. I like that it’s serves per person per day, because even when it is in [total] kilograms [purchased], it doesn’t translate well. What does a kilogram mean for a person on the ground”*

Store nutritionists had questions regarding what foods were included and excluded in certain food groups and subgroups; they indicated that it was important that the classification of foods as healthy or unhealthy aligned with their organisation’s nutrition policy and that misalignment could cause confusion. Examples of this were 100% juice being included in the fruit group when the messaging given to store managers was that juice is not healthy, as well as high-fibre white bread being classified with regular white bread as a “less healthy choice”, as store managers were instructed to stock high-fibre white bread varieties (that were fortified with other nutrients such as iron as well as fibre).

*“[Having high fibre white bread as less healthy is ]… a difficult one to explain to Store Managers particularly after they are told to stock Jackaroo and Bush Oven [high fibre white bread brands]…. which are more expensive products. Having the fortified white bread the same as white bread gives a store manager no incentive to stock it. Would better align with the organisation’s nutrition policy if included as a healthy choice.”*

Store group nutritionists indicated that it would be valuable to make monthly or quarterly reports available and that aggregated reports (combining multiple stores) would be valuable at the organisational level. They indicated the reports would be best integrated into their existing reporting system or disseminated via the store group nutritionists or store group retail area managers responsible for a group of stores. They would be best implemented through a train-the-trainer model where the nutritionists were trained in the background and use of reports, and they would provide training to managers in turn.

## 4. Discussion

Building from previous work that included extensive stakeholder consultation [12,13,19] and using data collected from a wide number of community stores, we developed and evaluated, through stakeholder feedback interviews, a reporting tool to support nutrition decision making of remote Aboriginal and Torres Strait Islander community stores. Key differences from this previous work are the use of a web-based reporting platform that allowed a wider choice of visuals and reporting comparator values alongside key dietary indicators, such as dietary guideline targets and average across all stores.

The strengths of the reports were the visual nature of the information provided, that they were easy to interpret, that they provided comprehensive information on a range of indicators and that they provided information that was easily linked to healthy diets (through reporting as serves per person per day and the inclusion of target values). The inclusion of comparator values alongside key dietary indicators was also highlighted as a strength of the reports by the stakeholders interviewed. Nutritionists indicated the potential value of the reports in increasing their capacity to perform their role. They also indicated that aggregated reports would be valuable at the organisational level, a function which could be added and made available to participating organisations.

While the stakeholders interviewed mostly indicated that the information in FoodFox reports fit with and added value to information already being received, store nutritionists indicated some misalignment of healthiness classification with other nutrition guidelines currently being used by the organisation or in the community. We based the classifications used in FoodFox reports on the Australian Dietary Guidelines, which use mostly a food-based rather than a nutrient profiling approach and are based on the best available evidence for healthy diets [27]. The Australian Dietary Guidelines Database enabled us to link products to food groups classified according to the Australian Dietary Guidelines. Despite this, we were challenged by the classification of some foods. One of these was high-fibre white bread, which is the most commonly purchased type of bread in remote Indigenous communities. The Australian Dietary Guidelines recommend wholegrains as the healthiest option and specify that refined grains are those with the bran and germ removed, even if fibre or other nutrients are added back, as these are unlikely to offer the same benefit as wholegrains [28]. The Australian Dietary Guideline Database groups breads containing >5 g fibre per 100 g together with wholemeal and wholegrain bread varieties based on what is used in the Australian Dietary Guideline modelling documents [26]. This made it difficult to determine whether the modified white bread product (which has 6–7 g fibre per 100 g) should be categorised in the healthiest category alongside wholegrain varieties or the less healthy category alongside regular white bread varieties. We ultimately chose the latter to be consistent with best evidence and broad public health messaging. One of the interviewed nutritionists also raised concerns over juice being categorised with fruit. Fruit juice was included in “less healthy” but still contributes to overall fruit estimates in line with the Australian Dietary Guidelines [27], although this is not without controversy [35]. This demonstrates the difficulty in defining what a healthy food is, which is an ongoing issue in nutrition promotion [35,36]. It is impossible to align perfectly with pre-existing classifications when they may differ based on relevance for different contexts, but for the most part, nutrition messaging in FoodFox reports is consistent with other messaging used in this setting.

Our method for disseminating the reports was not ideal, as evidenced by many of the store managers stating they had not looked at the reports prior to being contacted for feedback, as well as none having shared the information with their store board. Reasons cited for this were that there had not yet been a store board meeting, having recently commenced as manager at the store, or absence from the store due to leave. Store managers have a challenging role with many competing demands [19]. Brimblecombe et al. reported competing time pressures as a reason why store managers could not prioritise time for nutrition reports although they saw their value [12]. Other suggested methods for disseminating the reports to store managers and store boards were via the store group nutritionists or area managers, where they exist.

Public health nutritionists may be well placed in some cases to facilitate dissemination of reports to store boards in stores managed independently by a store group. Public health nutritionists are employed by government and non-government organisations to support remote communities to coordinate and implement nutrition promotion initiatives. They have, however, reported challenges in engaging effectively with store boards and managers to support health-promoting retail, with this being driven in part by lack of decision support tools and information. Day et al. (2023) found that providing these tools to public health nutritionists could enhance their capacity to work with stores, facilitating engagement with store staff, giving purpose to store visits and providing intelligence to guide their work [37]. We have shown in our previous research that providing reports to store boards in a facilitated way has stimulated rich discussion on health-enabling action the store could further take [12]. Another method we have found to be of benefit to the whole community is to firstly provide sales data reports to the store and then, with permission, use them with a community-level multi-sector food interest group to track changes in store sales alongside initiatives to improve population diet [13].

Our study contributes to the rapidly growing field of healthy food retail research through the development of a web-based tool that reports nutrition indicators from store sales data to remote store decision makers. The unique context of the communities we work with facilitates this work, including a strong sense of social purpose of store decision makers [13]. This research was made possible by a robust partnership between the researchers and community partners supported by prior research engagement and established agreements dictating the use and sharing of data, which may instil confidence in sharing potentially sensitive information. To our knowledge, no other tools have been specifically developed to provide such insights directly to store decision makers to inform store policy decisions to improve nutrition. However, there are similarities to other programs that measure and report metrics from retail data for nutrition-related purposes.

INFORMAS, the International Network for Food and Obesity/NCDs Research, Monitoring and Action Support, primarily focuses on monitoring, benchmarking and supporting public and private sector actions for obesity and non-communicable disease (NCD) prevention [4,5]. While INFORMAS’s primary goal is not to directly provide information to retailers, its activities indirectly inform retailers and other stakeholders in the food environment—for example, through benchmarking their retail policy and practice [4,6,7]. Retailers can utilise benchmarking data to compare their practices and offerings against national or international standards, pinpointing areas for improvement. Despite INFORMAS’s current set of indicators not including sales data, our web-based reporting tool aligns with the goals of INFORMAS by providing a systematic approach to reporting key dietary indicators designed to help build health-promoting food retail.

The Australian Bureau of Statistics uses sales data collected from major Australian supermarkets to provide valuable insights at a national level on diet quality, but does not aim to collect data that would have the granularity and community specificity for stores to tailor policy development to be relevant to their community [38]. By focusing on their store sales data, stores can better develop store policy and practice that addresses their purchasing patterns.

Limitations of this research include the fact that feedback on reports was not collected from all key stakeholder groups. Aboriginal and Torres Strait Islander store directors informed report development through community consultation; however, we did not collect direct feedback on the reports. Due to the high cost of travelling to remote communities in Australia, our plan was to seek store director feedback through interviews with the store managers; however, as the reports were not shared at store director meetings, this feedback was not available. Feedback from store directors on their experiences with using reports for decision making is necessary for understanding the usefulness of reports. We also did not capture the views of the public health nutritionists working in this setting, which was out of scope given the time that would have been required to gain the permission to share the reports owned by board members. Limitations of the reports developed are that they do not measure other aspects related to the consumer food environment, such as store practice or price, or suggestions for improvement to make the information readily actionable.

Future research directions include expanding the scope of the reporting tool to incorporate additional indicators related to consumer food environments, such as store practices, policy adoption and pricing. We are currently investigating whether reports of a wider range of indicators (including purchasing), as part of a benchmarking continuous improvement model, can support decision makers such as Aboriginal and Torres Strait Islander store board directors, retailers and nutritionists in developing effective store policy to support healthier customer purchases. Processing store sales data into nutrition indicators can be resource-intensive. Future research may explore opportunities for automation of data coding and checks to streamline this process, potentially improving the efficiency and scalability of such analyses. In the remote community store context, there is interest in internal ongoing monitoring and reporting of nutrition-related indicators to inform their organisation nutrition policies. There is potential for future research to explore solutions to integrate such nutrition reporting within their existing information systems. While larger store/supermarket companies are likely to have the capacity to build nutrition-related indicators into their point-of-sale monitoring and reporting systems, this may not occur without shareholder and/or public demand for such information.

## 5. Conclusions

We developed a web-based reporting tool of key dietary indicators from sales data to support health-promoting policy and practice in remote stores in Aboriginal and Torres Strait Islander communities. Store managers and store nutritionists indicated the strengths and potential value of these reports. Challenges were identified in aligning the healthiness classification used for key dietary indicators with the other nutrition guidelines used and ensuring reports reached relevant decision makers. This research builds on a comprehensive program of work and advances knowledge on how sales data can be used to provide nutrition-related information to store decision makers.

## Figures and Tables

**Table 1:** Population weighted dietary guideline targets used in FoodFox.

Food Group	Target (/Person/Day)
Fruit	≥2 serves
Vegetables	≥5 serves
Breads and Cereals	≥5.5 serves
Meat, Fish and Eggs	≥2.5 serves
Dairy	≥2.5 serves
Unhealthy Foods	≤2.5 serves

## Data Availability

Store sales, community consultation and stakeholder feedback data are not available due to ethical restrictions.

## Supplementary Materials

The following supporting information can be downloaded at https://www.mdpi.com/article/10.3390/nu16071058/s1. Supplement S1: FoodFox example report using mock data; Supplement S2: Explanatory materials.

## References

[1] World Health Organisation (2013). Global Action Plan for the Prevention and Control of Noncommunicable Diseases 2013–2020.

[2] Hartmann-Boyce J., Bianchi F., Piernas C., Riches S.P., Frie K., Nourse R., Jebb S.A. (2018). Grocery store interventions to change food purchasing behaviors: A systematic review of randomized controlled trials. Am. J. Clin. Nutr..

[3] Vogel C., Dijkstra C., Huitink M., Dhuria P., Poelman M.P., Mackenbach J.D., Crozier S., Seidell J., Baird J., Ball K. (2023). Real-life experiments in supermarkets to encourage healthy dietary-related behaviours: Opportunities, challenges and lessons learned. Int. J. Behav. Nutr. Phys. Act..

[4] Ni Mhurchu C., Vandevijvere S., Waterlander W., Thornton L.E., Kelly B., Cameron A.J., Snowdon W., Swinburn B., INFORMAS (2013). Monitoring the availability of healthy and unhealthy foods and non-alcoholic beverages in community and consumer retail food environments globally. Obes. Rev..

[5] Brinsden H., Lobstein T., Landon J., Kraak V., Sacks G., Kumanyika S., Swinburn B., Barquera S., Friel S., Hawkes C. (2013). Monitoring policy and actions on food environments: Rationale and outline of the INFORMAS policy engagement and communication strategies. Obes. Rev..

[6] Sacks G., Schultz S., Grigsby-Duffy L., Robinson E., Orellana L., Marshall J., Cameron A. (2020). Inside Our Supermarkets: Assessment of the Healthiness of Australian Supermarkets.

[7] Vandevijvere S., Waterlander W., Molloy J., Nattrass H., Swinburn B. (2018). Towards healthier supermarkets: A national study of in-store food availability, prominence and promotions in New Zealand. Eur. J. Clin. Nutr..

[8] Jenneson V.L., Pontin F., Greenwood D.C., Clarke G.P., Morris M.A. (2022). A systematic review of supermarket automated electronic sales data for population dietary surveillance. Nutr. Rev..

[9] Jenneson V., Greenwood D.C., Clarke G.P., Rains T., Tempest B., Shute B., Morris M.A. (2023). Supermarket Transaction Records In Dietary Evaluation: The STRIDE study: Validation against self-reported dietary intake. Public Health Nutr..

[10] Tin S.T., Mhurchu C.N., Bullen C. (2007). Supermarket sales data: Feasibility and applicability in population food and nutrition monitoring. Nutr. Rev..

[11] Brimblecombe J., Liddle R., O’Dea K. (2013). Use of point-of-sale data to assess food and nutrient quality in remote stores. Public Health Nutr..

[12] Brimblecombe J. Keeping Track of Healthy Foods: Towards Improving the Nutritional Quality of Foods Sold in Community Stores in Remote Australia; Menzies School of Health Research; Darwin, Australia, 2008.

[13] Brimblecombe J., Bailie R., van den Boogaard C., Wood B., Liberato S.C., Ferguson M., Coveney J., Jaenke R., Ritchie J. (2017). Feasibility of a novel participatory multi-sector continuous improvement approach to enhance food security in remote Indigenous Australian communities. SSM Popul. Health.

[14] Butler R., Tapsell L., Lyons-Wall P. (2011). Trends in purchasing patterns of sugar-sweetened water-based beverages in a remote Aboriginal community store following the implementation of a community-developed store nutrition policy. Nutr. Diet..

[15] Australian Bureau of Statistics 3238.0.55.001-Estimates of Aboriginal and Torres Strait Islander Australians, June 2016. https://www.abs.gov.au/ausstats/abs@.nsf/mf/3238.0.55.001.

[16] Outback Stores Outback Stores. https://outbackstores.com.au/about/health-nutrition/.

[17] The Arnhem Land Progress Aboriginal Corporation ALPA Health & Nutrition Strategy. https://www.alpa.asn.au/health-and-nutrition.

[18] Community Enterprise Queensland Health, Wellbeing & Nutrition. https://www.ceqld.org.au/health/.

[19] Rogers A., Ferguson M., Ritchie J., Van Den Boogaard C., Brimblecombe J. (2018). Strengthening food systems with remote Indigenous Australians: Stakeholders’ perspectives. Health Promot. Int..

[20] NHMRC (2013). Australian Dietary Guidelines.

[21] Greenacre L., van Burgel E., Hill A., McMahon E., Ferguson M., Rodrigues C., Brimblecombe J. Food Retail in Remote Australia. https://datadryad.org/stash/dataset/doi:10.5061/dryad.vmcvdnczc.

[22] The Arnhem Land Progress Aboriginal Corporation. https://www.alpa.asn.au/.

[23] Food Standards Australia New Zealand AUSNUT 2011–13—Australian Food Composition Database. www.foodstandards.gov.au.

[24] Food Standards Australia New Zealand Australian Health Survey—Australian Dietary Guidelines Database (Amended 3 January 2017). https://www.foodstandards.gov.au/science-data/monitoringnutrients/australianhealthsurveyandaustraliandietaryguidelines.

[25] Australian Bureau of Statistics (2014). 43630DO005_20112013 Australian Health Survey: User’s Guide, 2011-13_Discretionary Food List.

[26] NHMRC (2011). A Modelling System to Inform the Revision of the Australian Guide to Healthy Eating.

[27] NHMRC (2011). A Review of the Evidence to Address Targeted Questions to Inform the Revision of the Australian Dietary Guidelines.

[28] NHMRC Grain (Cereal) Foods, Mostly Wholegrain and/or High Cereal Fibre Varieties. https://www.eatforhealth.gov.au/food-essentials/five-food-groups/grain-cereal-foods-mostly-wholegrain-and-or-high-cereal-fibre.

[29] NHMRC Discretionary Food and Drink Choices. https://www.eatforhealth.gov.au/food-essentials/discretionary-food-and-drink-choices.

[30] Auerbach B.J., Wolf F.M., Hikida A., Vallila-Buchman P., Littman A., Thompson D., Louden D., Taber D.R., Krieger J. (2017). Fruit Juice and Change in BMI: A Meta-analysis. Pediatrics.

[31] Hebden L., O’Leary F., Rangan A., Singgih Lie E., Hirani V., Allman-Farinelli M. (2017). Fruit consumption and adiposity status in adults: A systematic review of current evidence. Crit. Rev. Food Sci. Nutr..

[32] (2016). Australian Bureau of Statistics. Australian Health Survey: Consumption of Food Groups from the Australian Dietary Guidelines. Australia 2011–12.

[33] Food Standards Australia New Zealand AUSNUT 2011–13. https://www.foodstandards.gov.au/science-data/food-nutrients-databases/ausnut-2011-13.

[34] Australian Bureau of Statistics (2014). Census 2011 Table Builder: Age in Single Years (AGEP), Sex (SEXP) and Indigenous Status (INGP), by Remoteness Area (RA) Counting: Persons, Place of Usual Residence.

[35] Jones A., Radholm K., Neal B. (2018). Defining ‘Unhealthy’: A Systematic Analysis of Alignment between the Australian Dietary Guidelines and the Health Star Rating System. Nutrients.

[36] Lobstein T., Davies S. (2009). Defining and labelling ‘healthy’ and ‘unhealthy’ food. Public Health Nutr..

[37] Day G., Collins J., Twohig C., De Silva K., Brimblecombe J. (2023). Towards healthy food retail: An assessment of public health nutrition workforce capacity to work with stores. Aust. N. Z. J. Public Health.

[38] Australian Bureau of Statistics Apparent Consumption of Selected Foodstuffs, Australia. https://www.abs.gov.au/statistics/health/health-conditions-and-risks/apparent-consumption-selected-foodstuffs-australia.

