# Supplementary material for: Development of a Tool for Reporting Key Dietary Indicators from Sales Data in Remote Australian Aboriginal and Torres Strait Islander Community Stores
Source: Nutrients. 2024 Apr 4;16(7):1058. doi: 10.3390/nu16071058 (PMC11013159; doi:10.3390/nu16071058)

FoodFox Report
Demonstration Store 1
July 2016 to June 2017

Menzies FoodFox reports are based on the information and food groupings from the *Australian Dietary Guidelines*.

Serves/person/day is the number of daily serves per person based on the estimated population. The population is estimated by dividing the total energy of foods and drinks sold by the average per person energy need (8.9MJ/person/day).

Average all stores is the average for all stores using FoodFox for that month/quarter.

Targets are the number of serves recommended to eat for health and wellbeing based on the *Australian Dietary Guidelines*.

Store goals are set by the user in the web system.

Serve size guide

Fruit

One serve fruit =

- 1 apple, banana, orange
- 2 apricots, kiwi fruit or plums
- 1 cup diced fruit
- 1/2 cup fruit juice
- 30g dried fruit

Breads & Cereals

One serve breads & cereals =

- 1 slice bread (40g)
- 1/2 medium roll or flat bread (40g)
- 1/2 cup cooked rice, pasta or noodles (75-120g)
- 1/2 cup cooked porridge (120g)
- 2/3 cup wheat cereal flakes (30g)
- 1/4 cup muesli (30g)
- 3 crackers or crispbreads (35g)
- 1 crumpet (60g) or a small English muffin or plain scone (35g)

Dairy

One serve dairy =

- 1 cup liquid milk (250ml)
- 4 tbs skim milk powder (25g)
- 1/2 cup evaporated milk (120ml)
- 2 slices hard cheese (40g)
- 1/2 cup ricotta cheese (120g)
- 3/4 cup yoghurt (200g tub)

Vegetables

One serve vegetables =

- 1/2 cup cooked vegetables
- 1 cup salad

Meat, Fish & Eggs

One serve meat, fish & eggs =

- 65g cooked red meat (90-100g raw)
- 80g cooked poultry (100g raw)
- 100g cooked fish (115g raw) or a small can of fish
- 2 large eggs (120g)
- 1 cup cooked/canned legumes (150g)
- 170g tofu
- 30g nuts or seeds

Unhealthy foods

One serve unhealthy foods =

- 1 can full sugar soft drink (375ml)
- 5-6 small lollies (40g)
- Small piece of plain cake (40g)
- 1/2 small bar chocolate (25g)
- 12 fried hot chips (60g)
- 1/4 meat pie (60g)
- 2-3 sweet biscuits (35g)
- 2 slices processed meats (50–60g)

Demonstration Store 1

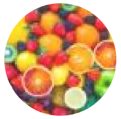

Fruit (serves/person/day)

Your Store June 2017

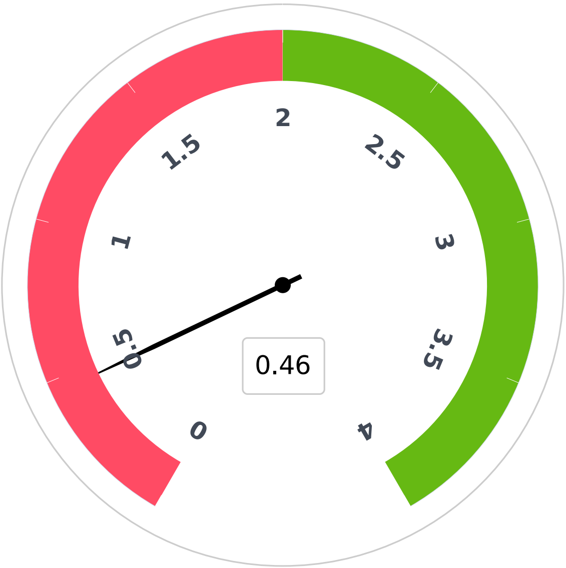

Fruit (serves/person/day)

- Below target < 2.00 serves/person/day
- Target zone ≥ 2.00 serves/person/day

Trend over time

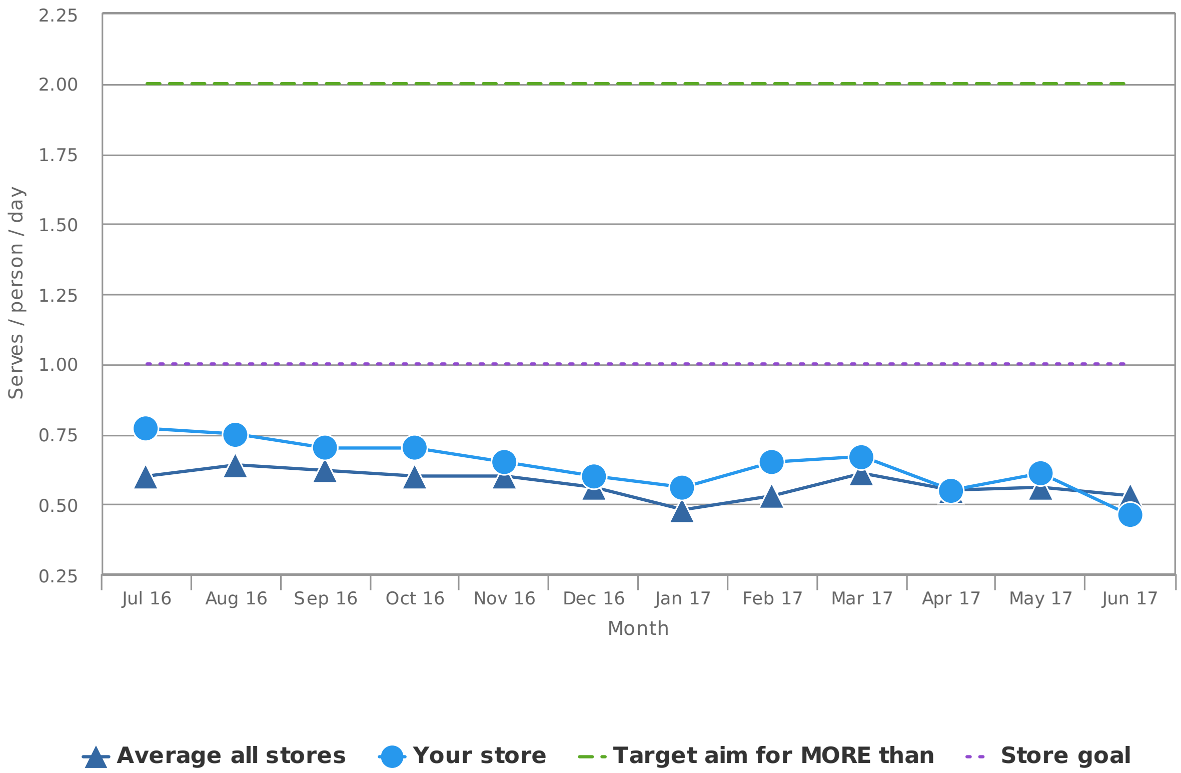

Demonstration Store 1

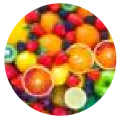

Types of Fruit (serves/person/day)

Your Store — June 2017

Best Choices

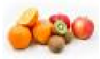

Fresh Fruit	0.21	serves

Less Healthy Choices

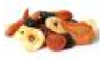

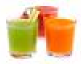

Dried Fruit	0.05	serves
Fruit Juice	0.20	serves

Trend over time

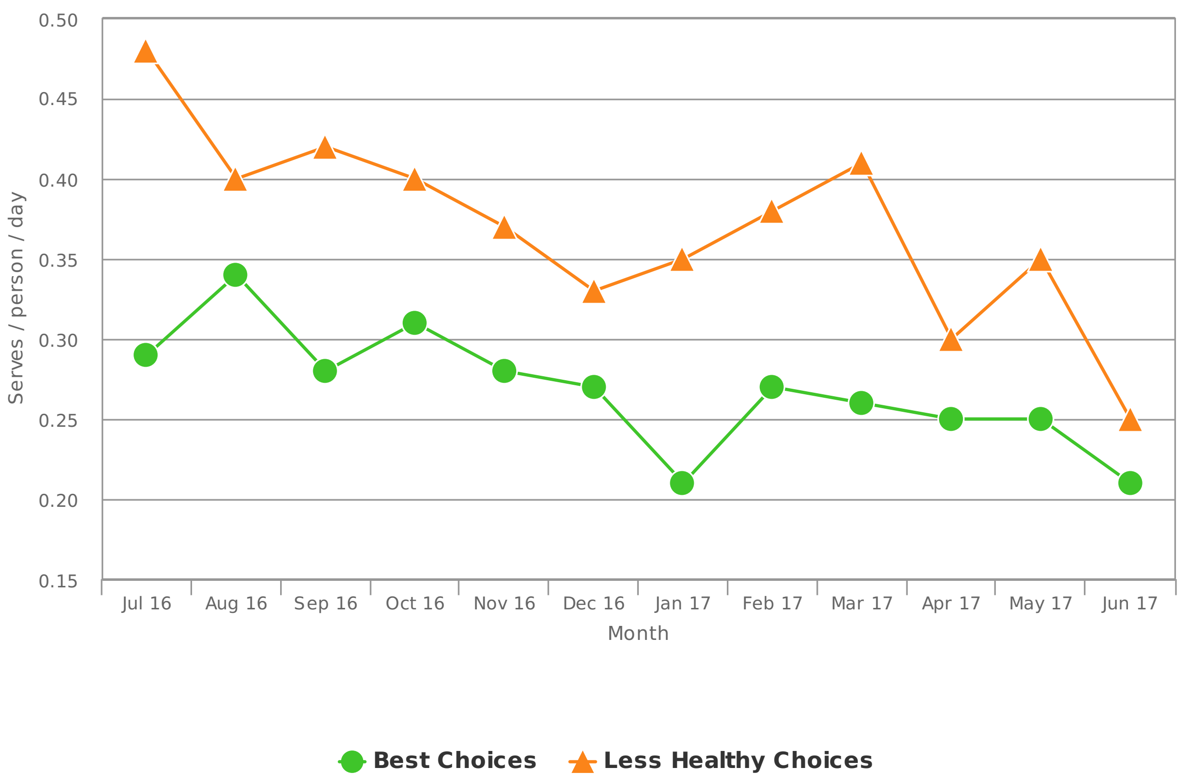

Demonstration Store 1

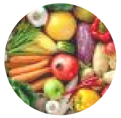

Vegetables (serves/person/day)

Your Store — June 2017

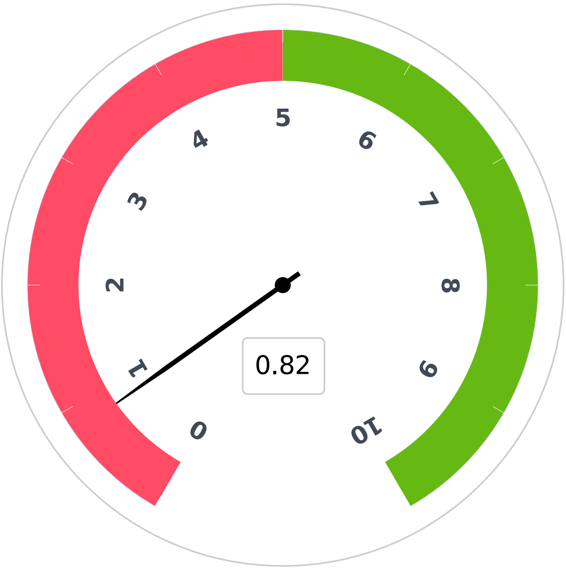

Vegetables (serves/person/day)

- **Below target** < 5.00 serves/person/day
- **Target zone** ≥ 5.00 serves/person/day

Trend over time

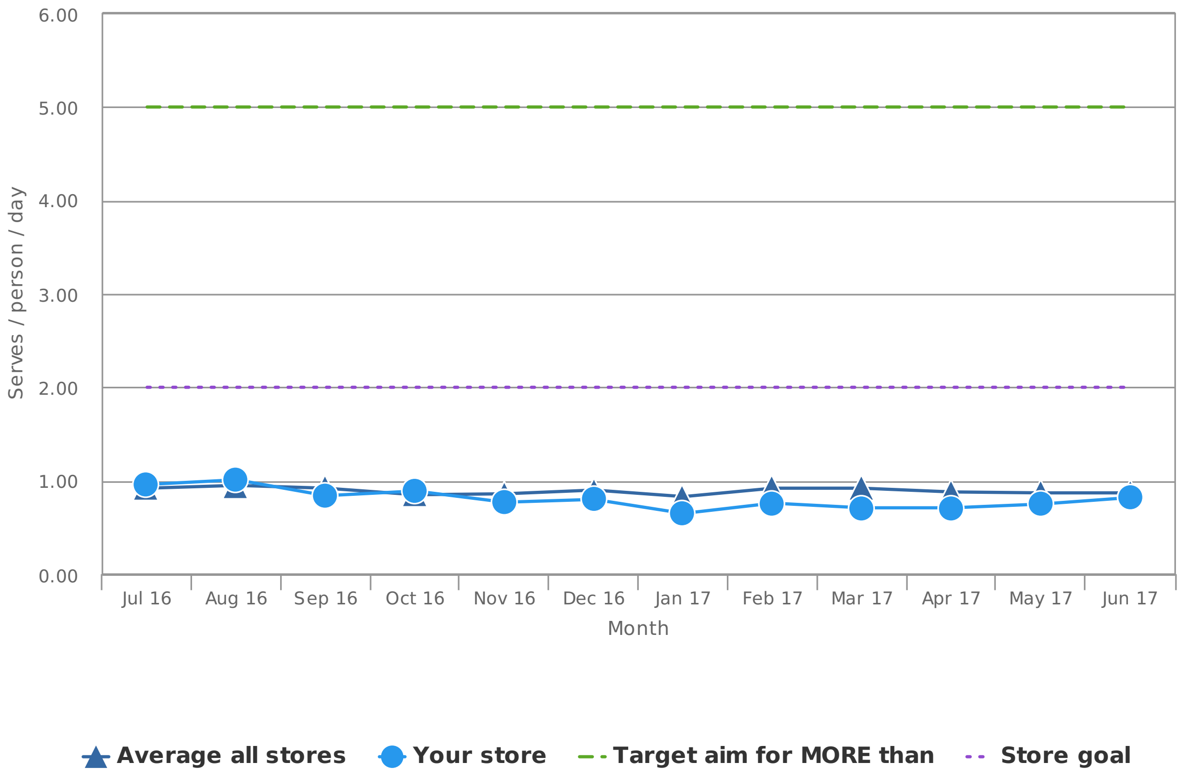

Types of Vegetables (serves/person/day)

Your Store — June 2017

Green Vegetables	0.11	serves	●
Orange Vegetables	0.08	serves	◆
Starchy Vegetables	0.21	serves	▲
Legumes	0.07	serves	■
Other Vegetables	0.35	serves	▼

Trend over time

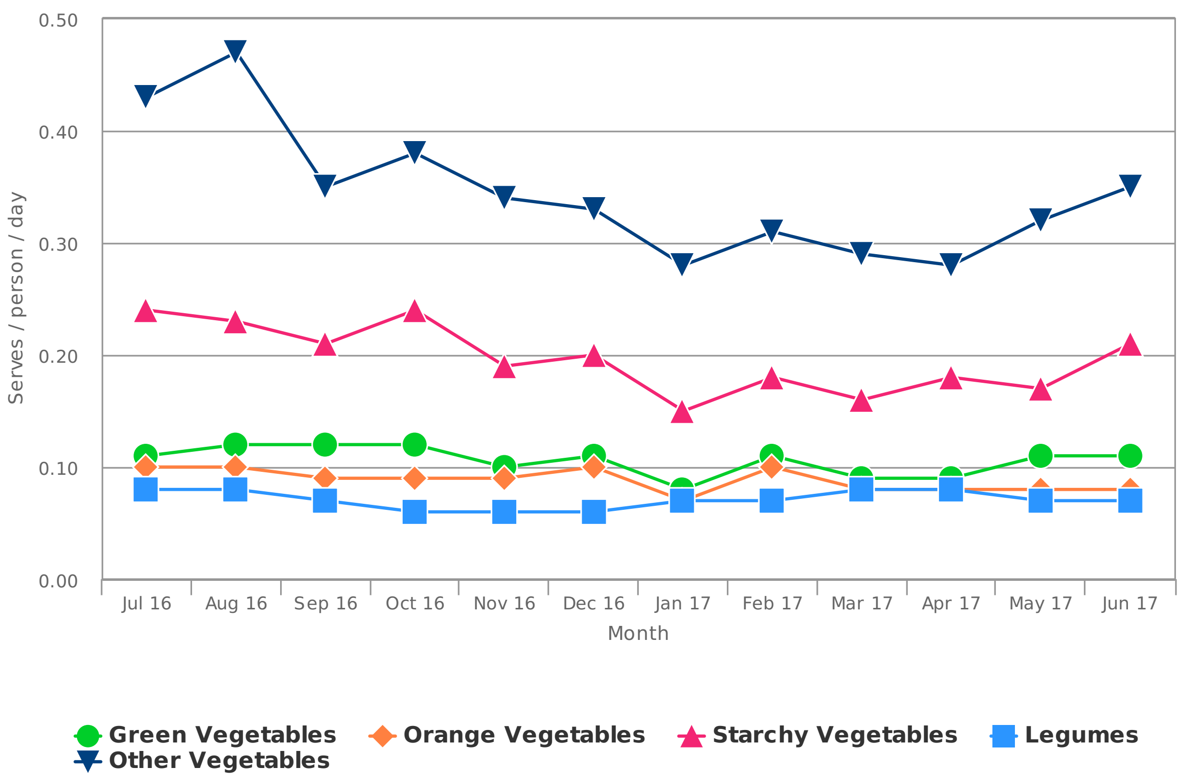

Demonstration Store 1

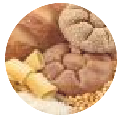

Breads & Cereals (serves/person/day)

Your Store June 2017

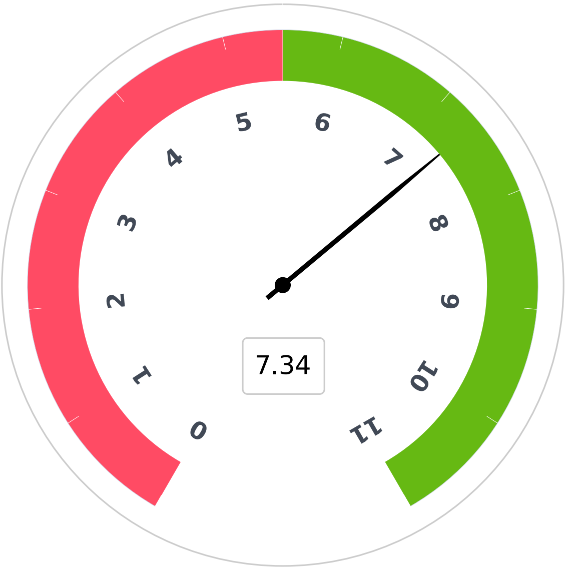

Breads & Cereals (serves/person/day)

● Below target < 5.50 serves/person/day

● Target zone ≥ 5.50 serves/person/day

Trend over time

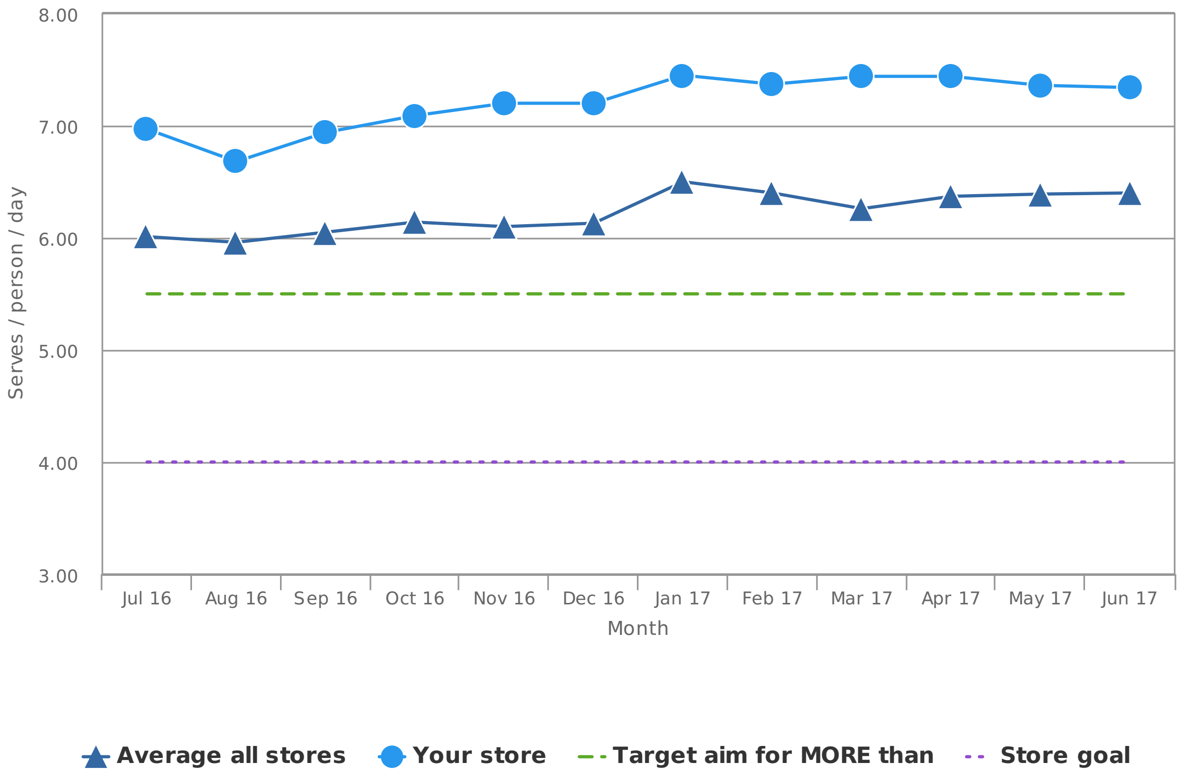

Types of Breads & Cereals (serves/person/day)

Your Store — June 2017

Best Choices

Wholegrain Bread	0.61	serves
Wholegrain Cereals	0.42	serves
Wholemeal Flour	0.00	serves
Other Wholegrains	0.01	serves

Less Healthy Choices

White Bread	3.15	serves
Refined Cereals	0.04	serves
White Flour	2.33	serves
Other Refined Grains	0.80	serves

Trend over time

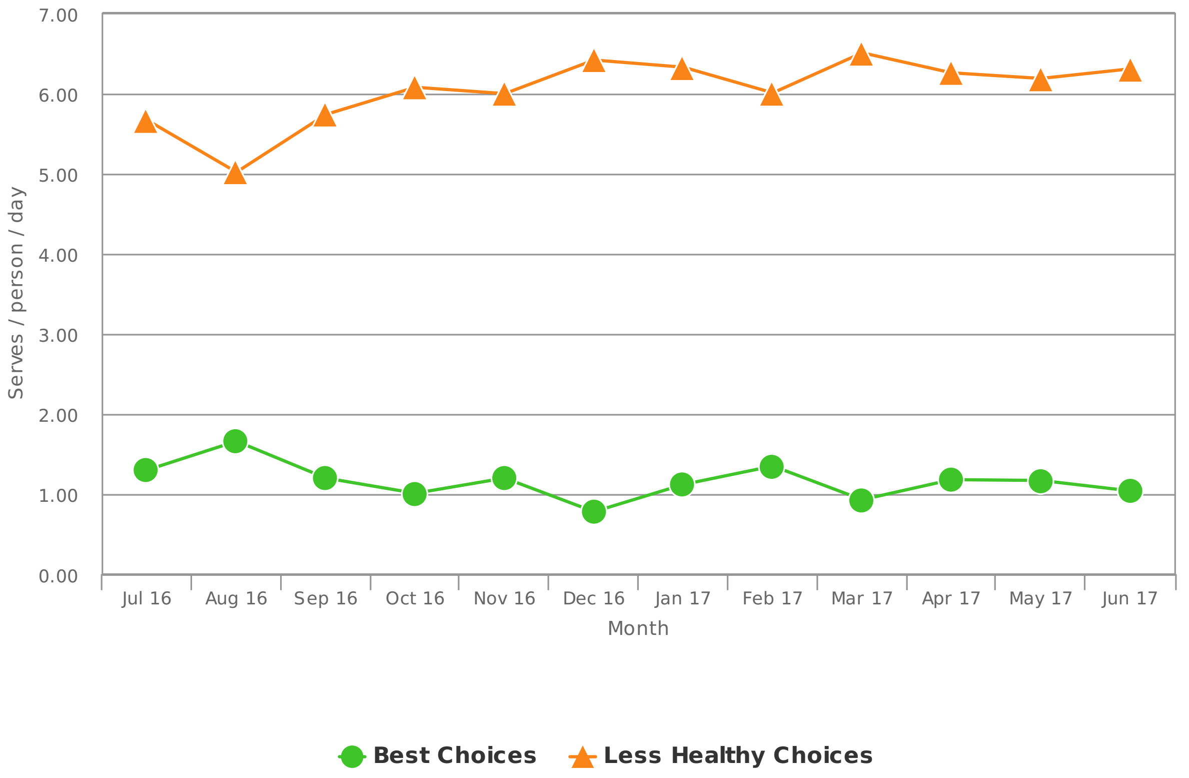

Demonstration Store 1

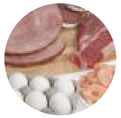

Meat, Fish & Eggs (serves/person/day)

Your Store — June 2017

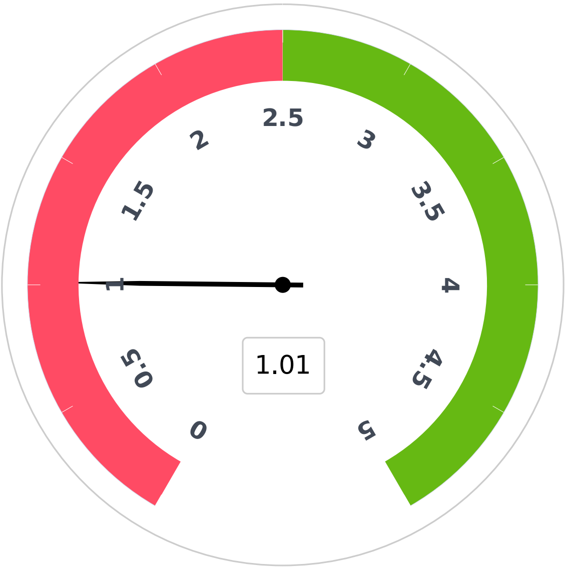

Meat, Fish & Eggs (serves/person/day)

- **Below target** < 2.50 serves/person/day
- **Target zone** ≥ 2.50 serves/person/day

Trend over time

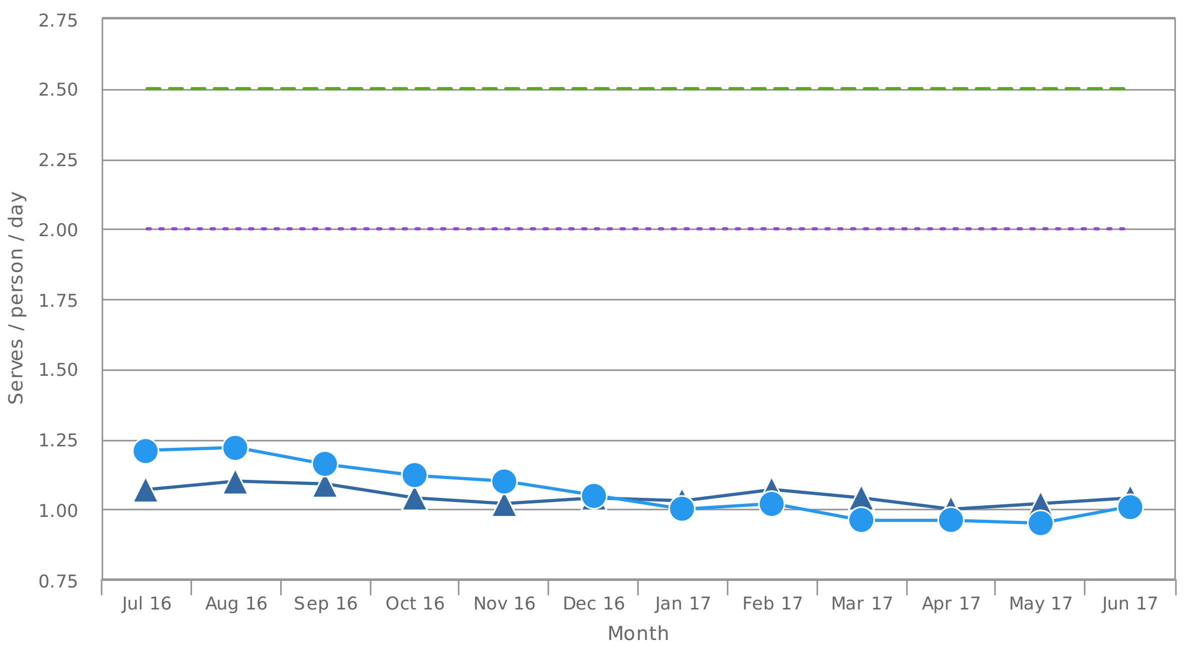

Average all stores **Your store** **Target aim for MORE than** **Store goal**

Types of Meat, Fish & Eggs (serves/person/day)

Your Store — June 2017

Best Choices

Lean Red Meat	0.13	serves
Lean Poultry	0.14	serves
Seafood	0.04	serves
Eggs	0.13	serves
Nuts & Legumes	0.13	serves

Less Healthy Choices

Not Lean Meat	0.39	serves

Trend over time

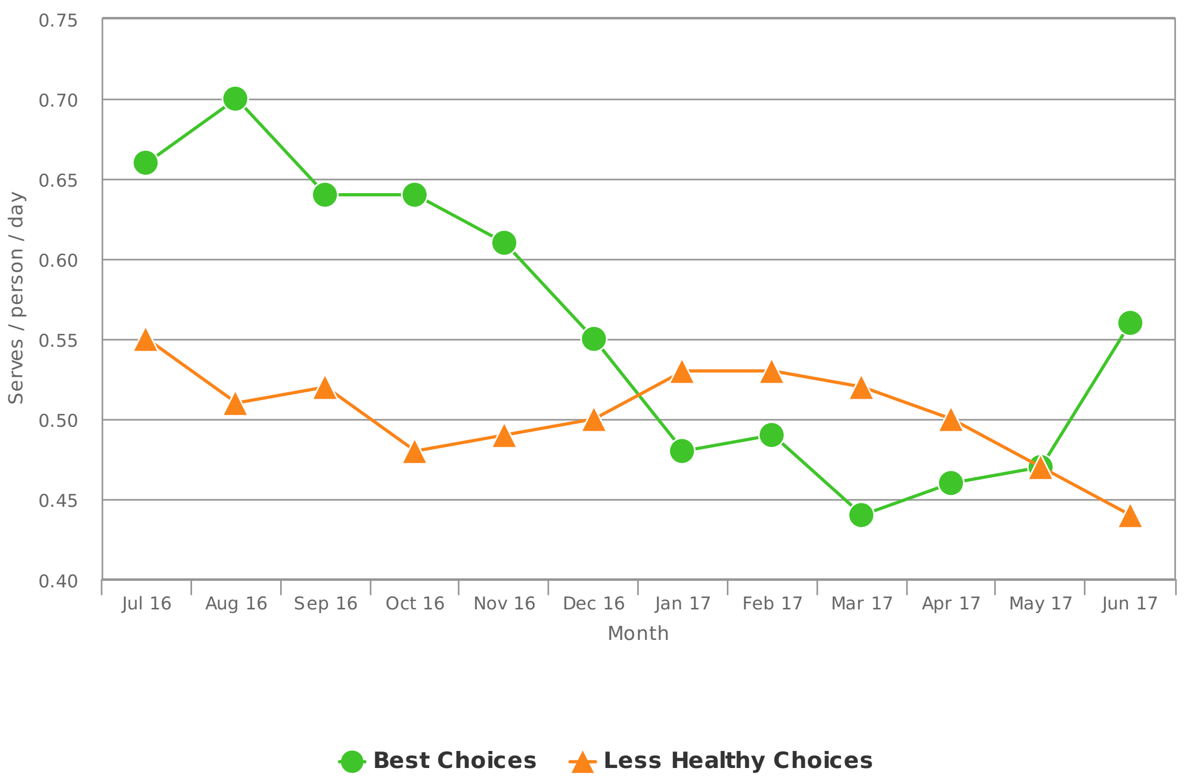

Demonstration Store 1

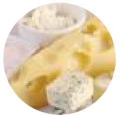

Dairy (serves/person/day)

Your Store June 2017

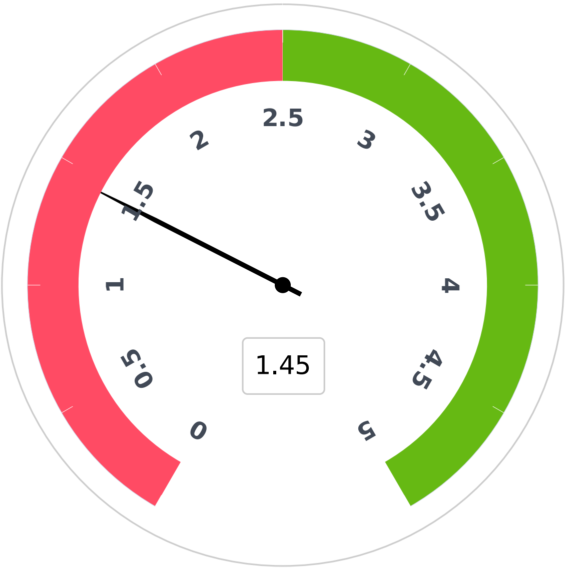

Dairy (serves/person/day)

- **Below target** < 2.50 serves/person/day
- **Target zone** ≥ 2.50 serves/person/day

Trend over time

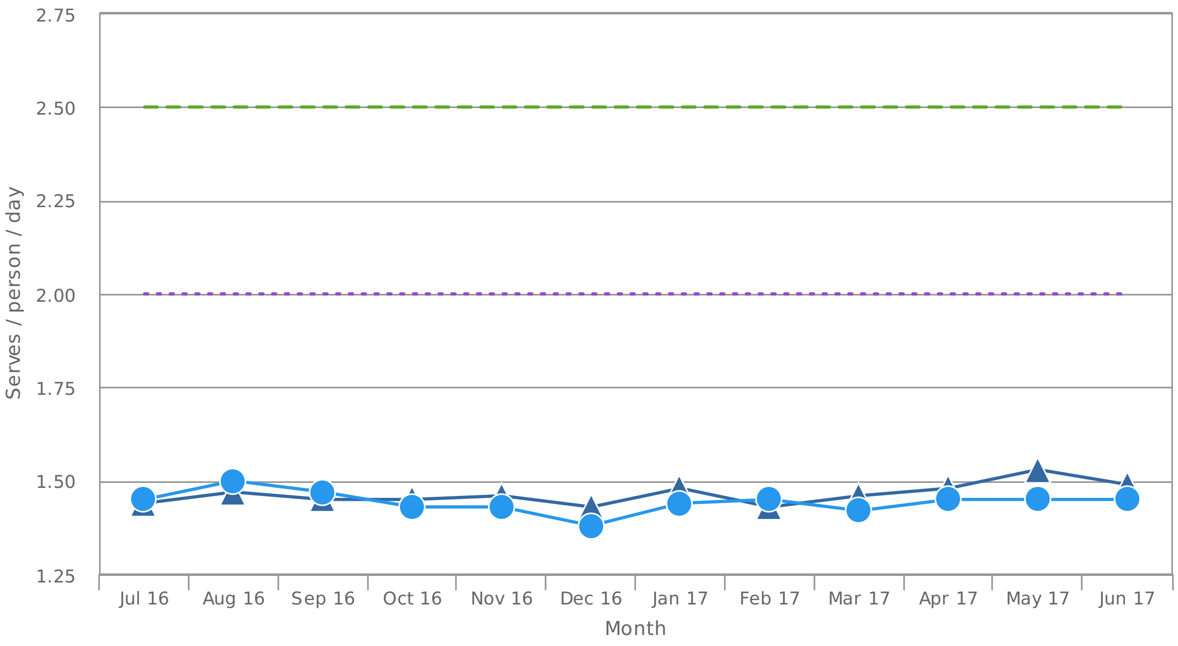

Average all stores **Your store** **Target aim for MORE than** **Store goal**

Demonstration Store 1

Types of Dairy (serves/person/day)

Your Store — June 2017

Best Choices

Reduced Fat Milk	0.02	serves
Reduced Fat Cheese	0.00	serves
Yoghurt	0.02	serves

Less Healthy Choices

Full Cream Milk	1.25	serves
Full Fat Cheese	0.16	serves

Trend over time

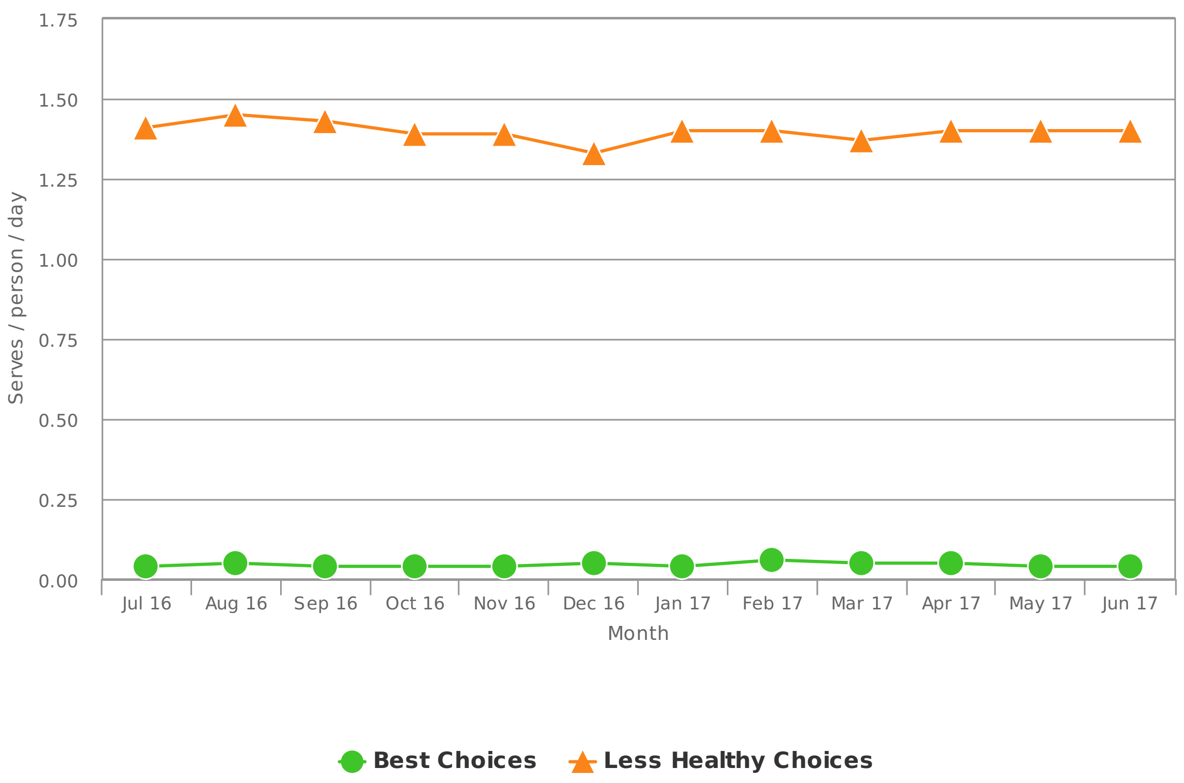

Demonstration Store 1

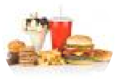

Unhealthy Foods (serves/person/day)

Your Store June 2017

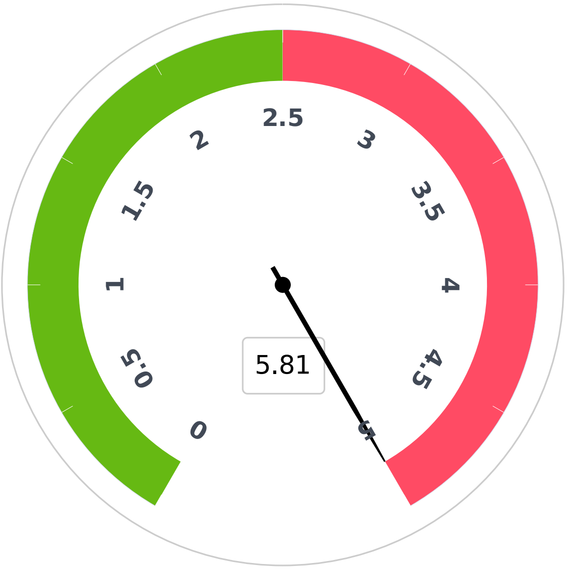

Unhealthy Foods (serves/person/day)

- **Below target** > 2.50 serves/person/day
- **Target zone** ≤ 2.50 serves/person/day

Trend over time

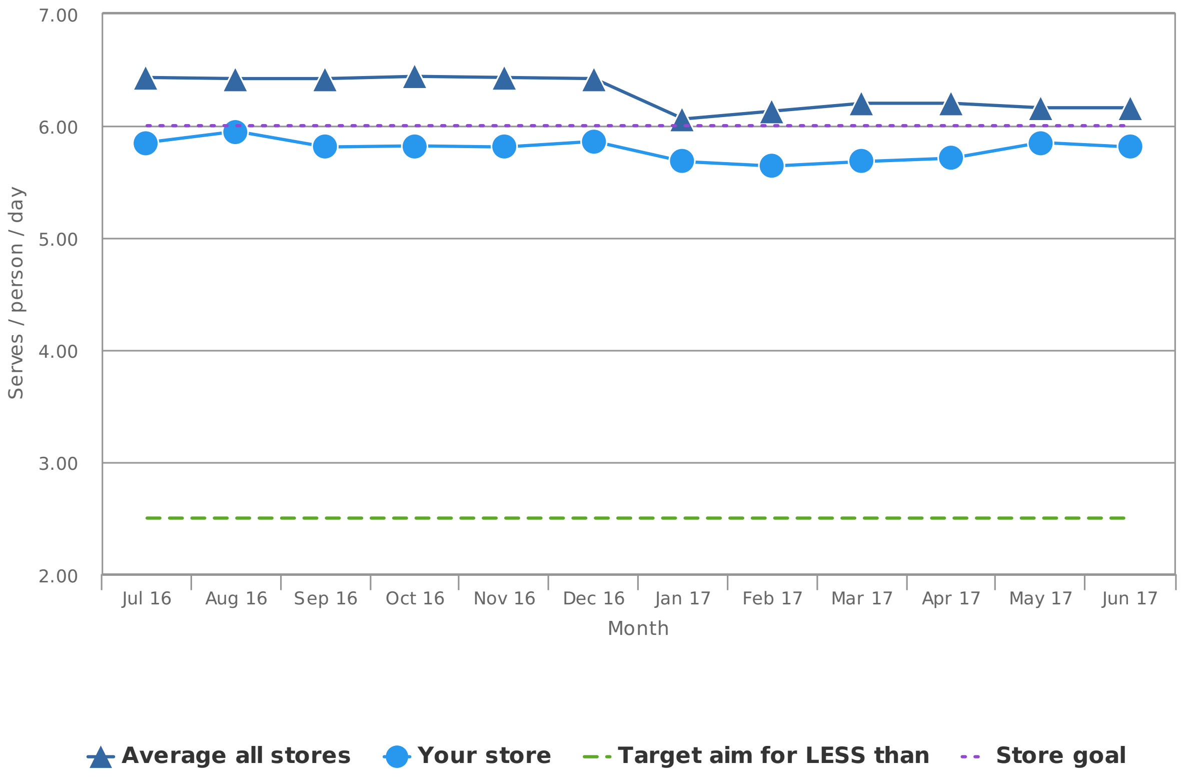

Demonstration Store 1

Types of Unhealthy Foods (serves/person/day)

Your Store — June 2017

Sugar	2.19	serves
Soft Drinks	0.77	serves
Biscuits	0.54	serves
Processed Meat	0.34	serves
Pies & Sausage Rolls	0.30	serves
Cordial	0.24	serves
Crisps	0.20	serves
Lollies	0.15	serves
Fruit Drinks	0.12	serves
Ice Cream & Ice Blocks	0.11	serves
Cake	0.10	serves
Chocolate	0.10	serves
Hot Chips	0.06	serves

Trend over time

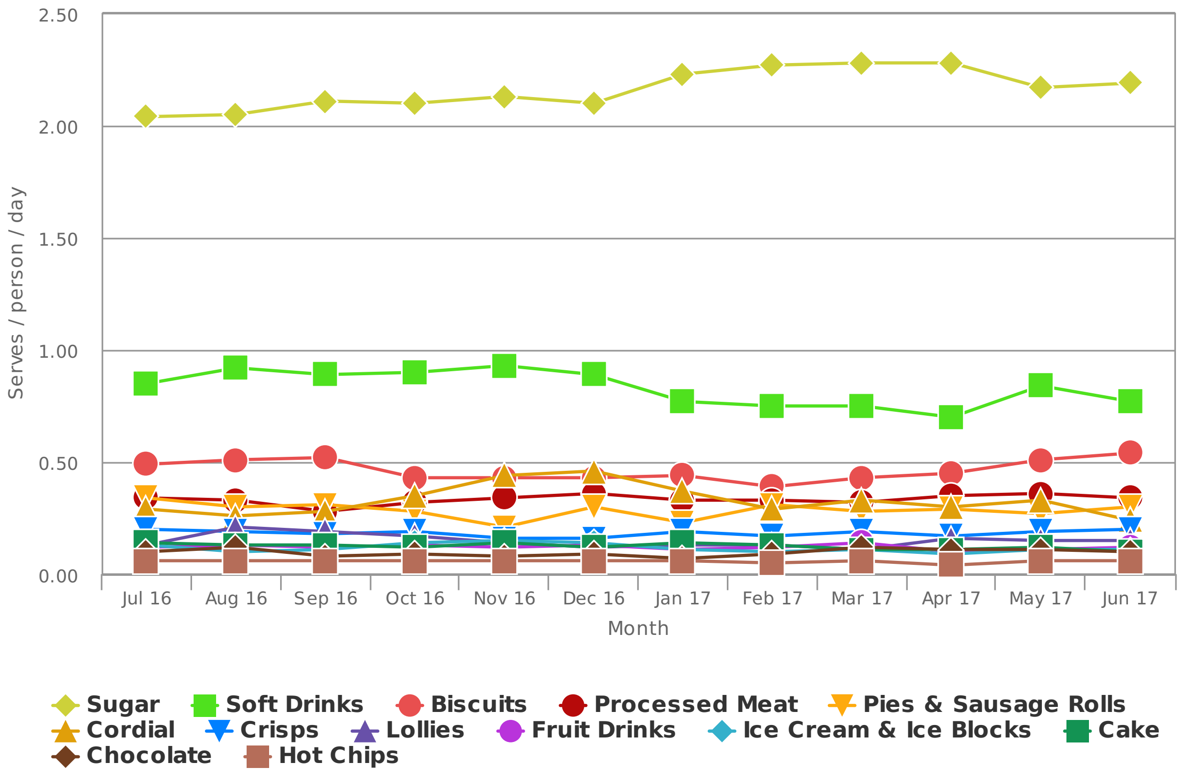

Supplement: Supplementary file 1 [file nutrients-16-01058-s001.zip › S1. FoodFoxDemoReport.pdf]
