# Supplementary material for: Development of a Tool for Reporting Key Dietary Indicators from Sales Data in Remote Australian Aboriginal and Torres Strait Islander Community Stores
Source: Nutrients. 2024 Apr 4;16(7):1058. doi: 10.3390/nu16071058 (PMC11013159; doi:10.3390/nu16071058)
Supplement: Supplementary file 1 [file nutrients-16-01058-s001.zip › S2. FoodFox_FAQs_serves_downloadable_v2.docx.pdf]

FAQs

What is FoodFox?

FoodFox translates electronic food and beverage sales data into food and nutrient information. The FoodFox web tool provides online & downloadable reports on the healthiness of foods sold in the store. This is based on the store sales data linked to food and nutrient data from the AUSNUT 2011-2013 and Australian Dietary Guidelines databases.

What do the reports show?

The reports show 'serves per person per day' for six food groups and types of food in these groups for your store compared to 'targets for good health' and the average of other participating stores.

How were food groups chosen?

Food groups are based on the Australian Dietary Guidelines, which show the types and amounts of foods people should eat to be healthy and prevent diseases like diabetes, heart disease and cancer. We also used these guidelines to categorise foods as being 'Best Choices' (●), 'Less Healthy Choices' (●) or 'Unhealthy Choices' (●). The table below gives more detail about what foods fall in each group.

What food group does mixed foods, like a sandwich, go in?

Some food products can go in more than one food group. For example, in an egg and salad sandwich, the bread part would be counted in Breads & Cereals (2 serves for 2 slices), the egg would be counted towards Meat, Fish & Eggs (1 serve for 2 eggs) and the salad would be counted in Vegetables (1 serve for 1 cup).

What does a 'serve' mean?

Serve sizes are those used in the Australian Dietary Guidelines and examples are given in the table below.

How is 'per person' calculated?

The per person values are calculated as the number of serves per 8.9 MJ energy (the average energy requirement per person per day). Using energy instead of population counts means that population movement doesn't affect the data as much.

How were store averages calculated?

The average of all stores is calculated by averaging all data from all stores for the selected time point.

How were targets chosen?

'Targets for good health' are based on the recommendations in the Australian Dietary Guidelines. These are given as minimum targets (aim for higher than) for all food groups except Unhealthy Foods, which are given as a maximum target (aim for lower than).

How is 'monthly' or 'quarterly' data calculated?

Data is usually given to us as weekly totals. This means the months often don't match up exactly with the calendar months. Months include all weeks where the last day of the data period is in that month (e.g. if the last day of the data was Sunday 1st of October, that week would be counted in the October data). This means that there can be 4 or 5 weeks in a month.

Why are some data points missing?

If we don't have all the sales data for a month or quarter, the data isn't shown on graphs. We also don't show the data if >5% of the products haven't been linked to their nutrition information (because we haven't finished the data processing yet).

FOODFOX GROUPINGS & SERVE SIZE GUIDE

(● BEST CHOICES ● LESS HEALTHY CHOICES ● UNHEALTHY CHOICES)

	SERVE GUIDE	SUBGROUP	INCLUDES
FRUIT	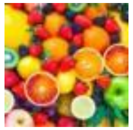<br>One serve (150g) =<br>• 1 apple, banana, orange<br>• 2 apricots, kiwi fruit or plums,<br>• 1 cup diced fruit<br>• 1/2 cup fruit juice<br>• 30g dried fruit	● **Fresh fruit** 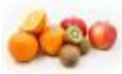	All fresh & frozen fruit as well as (drained) canned fruit
		● **Dried fruit** 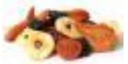	All dried fruit
		● **Fruit juice** 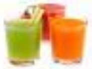	All 100% fruit juice
VEGETABLES	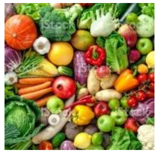<br>One serve (75g) =<br>• 1/2 cup cooked vegetables<br>• 1 cup salad	● **Green vegetables** 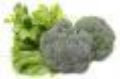	Cabbage, lettuce, broccoli, cauliflower, peas, green beans, asparagus, spinach
		● **Orange vegetables** 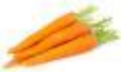	Pumpkin & carrot. Excludes sweet potato (included as starchy vegetable)
		● **Starchy vegetables** 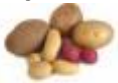	Potato, sweet potato, sweetcorn, cassava, taro
		● **Legumes** 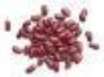	Lentils, kidney beans, chickpeas, soy beans, baked beans
		● **Other vegetables** 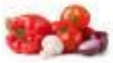	Tomato, onion, mushroom, avocado, garlic, beetroot, celery, cucumber, 100% vegetable juice

	SERVE GUIDE	SUBGROUP	INCLUDES
BREADS & CEREALS	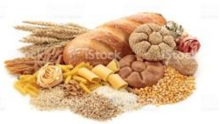<br>One serve (500kj) =<br>• 1 slice bread (40g)<br>• 1/2 medium roll or flat bread (40g)<br>• 1/2 cup cooked rice, pasta or noodles (75-120g)<br>• 1/2 cup cooked porridge (about 120g)<br>• 2/3 cup wheat cereal flakes (30g)<br>• 1/4 cup muesli (30g)<br>• 3 crackers or crispbreads (35g)<br>1 crumpet (60g) or a small English muffin or plain scone (35g)	● **Wholegrain bread** 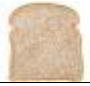	Wholegrain (WG) breads & bread rolls, WG flatbreads, wraps & pitas, WG crumpets, WG English muffins, WG scones (if low fat)
		● **Wholegrain cereals** 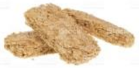	Wholegrain or high fibre cereals (≥5g dietary fibre), such as: wheat biscuits, oats, muesli, porridge, bran cereals
		● **Wholemeal flour** 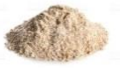	Wholemeal and wholegrain flours, such as: wholemeal wheat flour, spelt flour, rye flour, chickpea flour
		● **Other wholegrains** 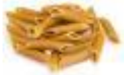	Wholegrain rice, noodles, pasta, crackers & other grains, such as: wholemeal pasta, wholemeal noodles, rye crackers, brown & wild rice, popcorn
		● **White bread** 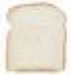	White bread & bread rolls, white flatbreads, wraps & pitas, white crumpets, white English muffins, white scones (if low fat)
		● **Refined cereals** 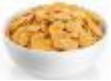	Refined, low fibre cereals (<5g dietary fibre), such as: Corn flakes, Special K, Nutrigrain, Puffed rice (e.g. Rice Bubbles)
		● **White flour** 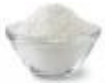	Refined flours, such as: white wheat flour (plain & self-raising), Cornflour, Rice flour, Arrowroot flour
		● **Other refined grains** 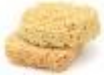	Refined grains or grain products such as: white pasta & rice, instant noodles, egg noodles, rice crackers, white wheat crackers (e.g.water crackers), couscous, tapioca

	SERVE GUIDE	SUBGROUP	INCLUDES
MEAT, FISH & EGGS	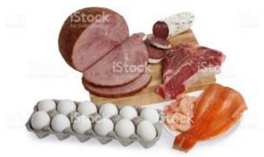<br>One serve (500-600kj) =<br>• 65g cooked red meat (90-100g raw)<br>• 80g cooked poultry (100g raw)<br>• 100g cooked fish (115g raw) or a small can of fish<br>• 2 large eggs (120g)<br>• 1 cup cooked/canned legumes (150g)<br>• 170g tofu<br>• 30g nuts or seeds	● **Lean red meat** 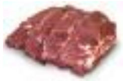	Red meat with < 10% fat, such as: beef, lamb, kangaroo, pork, veal, lean, lower salt sausages
		● **Lean poultry** 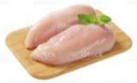	Poultry with < 10% fat, such as: chicken, duck, emu, goose, turkey
		● **Seafood** 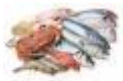	All fish & seafood, such as: fish, clams, crab, lobster, mussels, oysters, prawns, scallops
		● **Eggs** 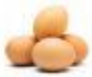	All eggs such as chicken or duck eggs
		● **Nuts & legumes** 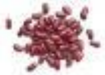	Nuts & seeds, nut/seed pastes (e.g. peanut butter, tahini), canned/dried legumes, tofu
		● **Not lean meat** 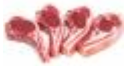	Poultry & red meat with ≥10% fat
DAIRY	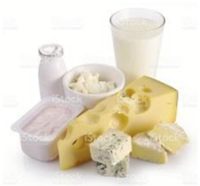<br>One serve (500-600kj) =<br>• 1 cup liquid milk (250ml)<br>• 4 tbs skim milk powder (25g)<br>• 1/2 cup evaporated milk (120ml)<br>• 2 slices hard cheese (40g)<br>• 1/2 cup ricotta cheese (120g)<br>• 3/4 cup yoghurt (200g tub)	● **Reduced fat milk** 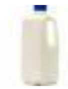	Dairy & non-dairy milks with <4% fat, such as: skim milk powder, trim/skim milk, reduced fat flavoured milk
		● **Reduced fat cheese** 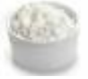	Cheeses with ≤ 10% fat, such as: reduced fat cheddar cheese & cream cheese, cottage cheese, ricotta cheese
		● **Yoghurt** 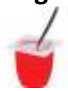	All yoghurts (all fat levels)
		● **Full cream milk** 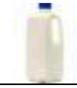	Dairy & non-dairy milks with ≥4% fat, such as: full cream milk powder, full cream milk, regular fat flavoured milk
		● **Full fat cheese** 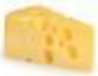	Cheeses with >10% fat, such as: full fat cheddar cheese & cream cheese, cheese spread, fetta, haloumi, mozzarella, soft cheeses (e.g. brie, camembert)

	SERVE GUIDE	SUBGROUP	INCLUDES	SUBGROUP	INCLUDES
UNHEALTHY FOODS	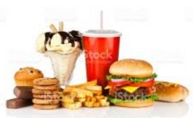<br>One serve (600kj) =<br>• 1 can full sugar soft drink (375ml)<br>• 5-6 small lollies (40g)<br>• Small piece of plain cake (40g)<br>• 1/2 small bar chocolate (25g)<br>• 12 fried hot chips (60g)<br>• 1/4 meat pie (60g)<br>• 2-3 sweet biscuits (35g)<br>• 2 slices processed meats (50–60g)<br>• 1/6 cup sugar (35g)	**Sugar**<br>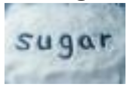	White sugar, brown sugar, icing sugar, raw sugar	**Cake**<br>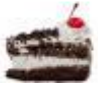	Cakes, cupcakes, slices, muffins, banana bread, scones (if high fat or sugar)
		**Soft drinks**<br>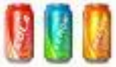	All soft drinks: cola drinks, iced teas, energy drinks	**Hot chips**<br>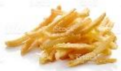	Hot chips/fries, potato gems, potato wedges, potato scallops, hash browns
		**Processed meat**<br>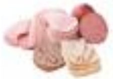	Canned corn beef, bacon, sausages, ham & other deli meats	**Ice cream/blocks**<br>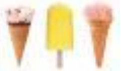	Includes all ice-blocks, ice-creams, sorbets & frozen yoghurts
		**Pies & sausage rolls**<br>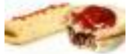	All savoury pastry products, such as: meat pies, sausage rolls, spring rolls, dim sims, samosas, wontons	**Crisps**<br>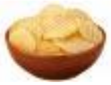	All savoury crisps made from potato, other vegetables or grains, such as: potato crisps, wheat crisps (e.g. Grain waves), corn chips, extruded or puffed savoury snacks (e.g. Cheese puffs)
		**Biscuits**<br>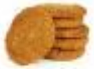	All sweet biscuits and wafers (including ice cream cones)		
		**Lollies**<br>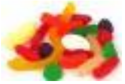	All lollies & non-chocolate confectionery, such as: jelly & boiled lollies, chewing gum, sherbet, lollipops, liquorice, marshmallow, honeycomb, fudge	**Cordial**<br>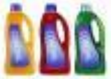	All cordials & cordial bases
		**Chocolate**<br>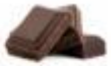	All chocolate & carob based confectioneries, such as: chocolate bars & blocks, chocolate bullets, chocolate-coated almonds, chocolate sultanas, rocky road	**Fruit drinks**<br>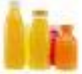	All fruit drinks (ready to drink & concentrates)

Document modified 6/7/2018
